# Supplementary material for: Role of Staphylococcus aureus’s Buoyant Density in the Development of Biofilm Associated Antibiotic Susceptibility
Source: Microorganisms. 2024 Apr 9;12(4):759. doi: 10.3390/microorganisms12040759 (PMC11052065; doi:10.3390/microorganisms12040759)
Supplement: Supplementary file 1 [file microorganisms-12-00759-s001.zip › microorganisms-2931583-supplementary.pdf]

Supplementary Material

Role of *Staphylococcus aureus*'s Buoyant Density in the Development of Biofilm Associated Antibiotic Susceptibility

Sarah Kispert[1,†], Madison Liguori[1, †], Cody Velikaneye[1], Chong Qiu[1], Shue Wang[1], Nan Zhang[2], Huan Gu[1,*]

[1]Department of Chemistry & Chemical Engineering and Biomedical Engineering, Tagliatela College of Engineering, University of New Haven, West Haven, CT 06516, United States

[2]Department of Pharmaceutics and Key Laboratory of Targeting Therapy and Diagnosis for Critical Diseases for Critical Diseases of Henan Province, School of Pharmaceutical Sciences, Zhengzhou University, Henan 450001, PR China

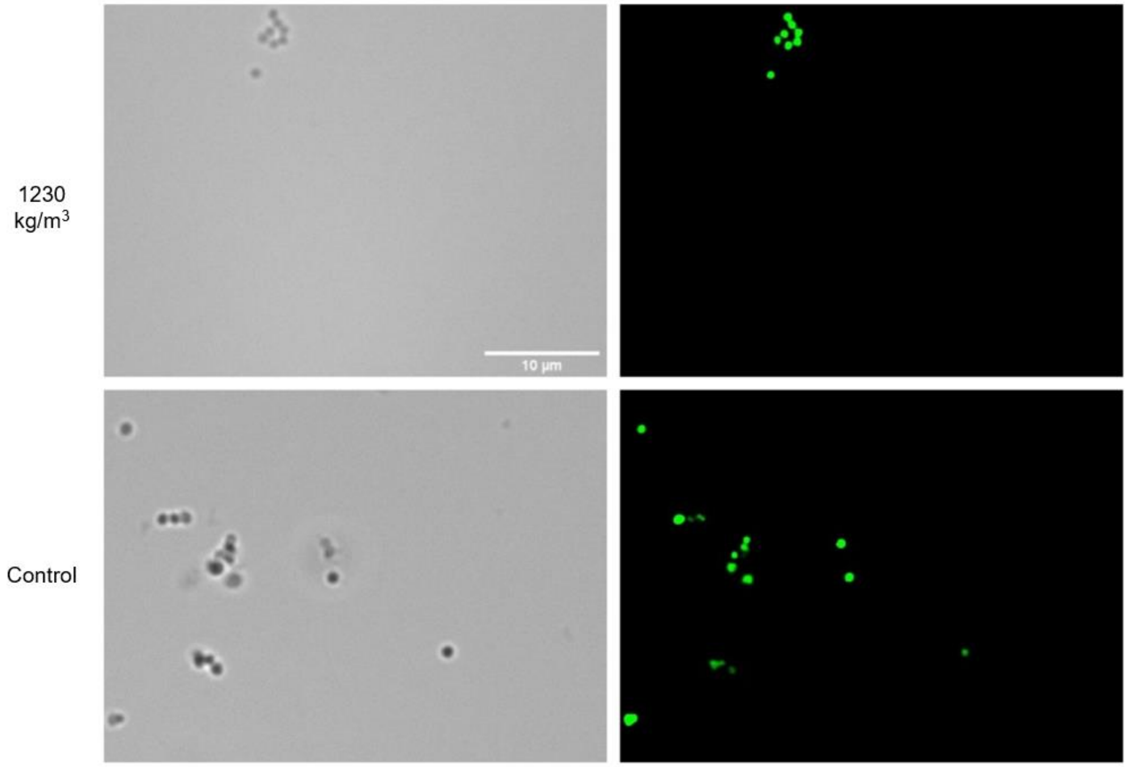

Figure S1. DNA content of cells for 18-hour culture. Zoomed in images of Brightfield and green fluorescence channels for *S. aureus* cells with the density of 1,230.00 kg/m^3. The zoomed images of mixed *S. aureus* cells before separation are included as controls (Bar = 10 μm).
